# Second generation sequencing allows for mtDNA mixture deconvolution and high resolution detection of heteroplasmy

**DOI:** 10.3325/cmj.2011.52.299

**Published:** 2011-06

**Authors:** Mitchell M. Holland, Megan R. McQuillan, Katherine A. O’Hanlon

**Affiliations:** 1 Forensic Science Program, The Pennsylvania State University, University Park, Pa, USA; 2 Oakland County Sheriff's Office Forensic Laboratory, Pontiac, Mich, USA

## Abstract

**Aim:**

To use parallel array pyrosequencing to deconvolute mixtures of mitochondrial DNA (mtDNA) sequence and provide high resolution analysis of mtDNA heteroplasmy.

**Methods:**

The hypervariable segment 1 (HV1) of the mtDNA control region was analyzed from 30 individuals using the 454 GS Junior instrument. Mock mixtures were used to evaluate the system’s ability to deconvolute mixtures and to reliably detect heteroplasmy, including heteroplasmic differences between 5 family members of the same maternal lineage. Amplicon sequencing was performed on polymerase chain reaction (PCR) products generated with primers that included multiplex identifiers (MID) and adaptors for pyrosequencing. Data analysis was performed using Next*GENe*^®^ software. The analysis of an autosomal short tandem repeat (STR) locus (D18S51) and a Y-STR locus (DYS389 I/II) was performed simultaneously with a portion of HV1 to illustrate that multiplexing can encompass different markers of forensic interest.

**Results:**

Mixtures, including heteroplasmic variants, can be detected routinely down to a component ratio of 1:250 (20 minor variant copies with a coverage rate of 5000 sequences) and can be readily detected down to 1:1000 (0.1%) with expanded coverage. Amplicon sequences from D18S51, DYS389 I/II, and the second half of HV1 were successfully partitioned and analyzed.

**Conclusions:**

The ability to routinely deconvolute mtDNA mixtures down to a level of 1:250 allows for high resolution analysis of mtDNA heteroplasmy, and for differentiation of individuals from the same maternal lineage. The pyrosequencing approach results in poor resolution of homopolymeric sequences, and PCR/sequencing artifacts require a filtering mechanism similar to that for STR stutter and spectral bleed through. In addition, chimeric sequences from jumping PCR must be addressed to make the method operational.

The year 2011 marks the 20th anniversary of the use of mitochondrial DNA (mtDNA) sequence analysis in forensic identification cases by the Armed Forces DNA Identification Laboratory (AFDIL), and the 15th anniversary of the introduction of mtDNA findings in a United States court of law by the Federal Bureau of Investigation (FBI) Laboratory (Paul William Ware v. State of Tennessee). While the proposition of using mtDNA analysis in casework dates back to the late 1980s (1), methods and initial forensic guidelines were developed in the early 1990s, leveraging the work of the ancient DNA community (2-6). Characteristics of mtDNA biology were investigated throughout the 1990s, which led to a better understanding of population diversity, mtDNA mutation rates, and the existence and prevalence of mtDNA heteroplasmy (7-16). In addition, the forensic targets for mtDNA analysis expanded to evidence such as crab lice, canines, and entomological specimens (17-19). Since that first decade, advanced guidelines for forensic mtDNA analysis have been published (20-22), whole genome sequencing approaches have been developed (23), and movements have been made toward analysis of mtDNA at the genome level (24). Therefore, the use of mtDNA testing to answer questions in forensic investigations is well established, and continues to provide forensic practitioners with a valuable analytical tool.

Historically, the principal targets for human mtDNA sequence analysis have been the two hypervariable segments of the control region; HV1 and HV2 (2). Based on the numbering system of Anderson et al (25), HV1 encompasses positions 16 024-16 365 and HV2 covers positions 73-340. While considerable work has been done to query other segments of the control region (26,27), as well as strategic positions throughout the entire genome (28,29), the vast majority of the discrimination potential is found in HV1 and HV2. Segments of the control region between 16 365-16,569, 1-72, and 341-573 have revealed both single nucleotide polymorphisms (SNP) and length-based variation, and specific wobble positions of the coding region possess polymorphic characteristics. Combined, these regions contain a reasonable amount of additional discrimination power, yet their analysis can require considerable additional effort.

The current methods of choice for performing routine forensic mtDNA sequence analysis are polymerase chain reaction (PCR) amplification of HV1 and HV2 followed by the dideoxynucleotide chain-termination sequencing technique of Sanger (30), and the use of capillary electrophoresis to analyze the fluorescently tagged products (31). The simultaneous sequencing of a pool of PCR amplicons generated from a DNA extract produces a profile of the predominant component found in the amplicon population. Minor components of an mtDNA mixture, detected at a level of 5%-20% depending on the analytical noise in the DNA sequencing instrument, are typically observed as a superimposition of two nucleotides at the same sequence position or a repetitive series of nucleotide residues of various lengths. Each of these heteroplasmic types can be difficult to interpret due to the overlapping nature of the sequence information, and the sequence of length variants downstream of the homopolymeric segment can be uninterpretable.

Mixtures of mtDNA sequence come from a combination of two or more different sources of DNA, or can arise from individuals exhibiting heteroplasmy. The existence of heteroplasmy, a **hetero**geneous pool of mtDNA sequences in the mitochondria found in the cyto**plasm** of cells from a single individual, was first reported in 1995 (12), and has since been studied quite extensively over the last 16 years (32-36); including the use of alternative techniques for detection of heteroplasmic variants (37-41). Of particular interest, and excitement, is the potential use of second generation sequencing (SGS) approaches for the deconvolution of mtDNA mixtures, including the resolution of heteroplasmic variants (42-46).

Of the available SGS technologies (eg, the 454 Life Science FLX from Roche, the SOLiD 4 from Applied Biosytems, the HiSeq 2000 from Illumina, and the Heliscope Single Molecule Sequencer from Helicos), the one that may best suit targeted sequencing of forensic loci is the 454 Life Sciences system, as it can directly sequence amplicons of 400-500 bps in length (47,48). Roche has recently released a small bench-top instrument (the GS Junior) which is more accessible to forensic laboratories, as the technology costs less than US $100 000 to purchase, and the cost per run is less than US $1000. The sequencing chemistry is based on a pyrosequencing approach (49). Nucleotides flow sequentially across a picotiter plate containing ~ 100 000 reaction wells ( ~ 1/10th the capacity of the larger 454 FLX instrument). Each well is ~ 44 μm in diameter and will accommodate a single DNA capture bead of ~ 28 μm coated with amplified copies of a single original amplicon. The second round of amplification occurs through a unique emulsion PCR (emPCR) technique, where each bead attaches to a single amplicon molecule, with the emPCR occurring in an aqueous micro-droplet containing the PCR reagents and surrounded by an oil-based emulsion. During the pyrosequencing, if a nucleotide is incorporated during the sequencing process, a coupled reaction between sulfurylase and luciferase will generate photons of light that can be captured by a charged-coupled device (CCD) camera. When multiple nucleotides are incorporated into a homopolymeric stretch, the signal intensity increases, and is proportional to the total number of incorporations. The pyrosequencing approach and the GS Junior technology allows for the parallel sequencing of ~ 100 000 DNA fragments in a single 8-hour run, or is comparable to running approximately one thousand 96-well plates of Sanger sequencing simultaneously. The result is a level of sequencing depth only seen in cloning experiments, at a small fraction of the cost and time necessary to complete such a study. Therefore, it is clear that the SGS technique from 454 Life Sciences has a potential place in forensic investigations.

Thus far, mtDNA sequence analysis has been used primarily in criminal cases involving hair shafts from humans and animals, and in cases of identification when skeletal material is available or when appropriate references are missing for conventional STR analysis. In a relatively high percentage of these cases, the presence of mtDNA heteroplasmy (both single nucleotide and length-based) can complicate profile interpretation. In a much larger percentage of cases, the heteroplasmic information is lost due to limitations of the traditional dideoxy-Sanger DNA sequencing method. In addition, mixtures of mtDNA sequences have historically not been reported or considered, significantly lowering the percentage of cases where mtDNA analysis can be, and is applied. When complex issues of heteroplasmy or mixtures arise, cloning experiments can often times resolve the questions (11), but these studies are quite labor intensive, costly, and impractical. Therefore, the purpose of the research reported here was to explore the potential for using an SGS approach to enhance the detection and reportability of mtDNA heteroplasmic variants, and for the deconvolution of mtDNA mixtures. Newly developed and validated methods for such analysis should increase the discrimination potential of the mtDNA typing system, and could be more cost-effective than traditional methods currently in practice. In addition, if the method proves to be robust, it may be possible to differentiate between maternal relatives through the analysis of low-level, de novo heteroplasmic variants. Lastly, the SGS approach was assessed for its ability to perform STR analysis, including when STR loci are multiplexed with the analysis of Y-STR and mtDNA loci. The preliminary results from this study were reported at the 20th International Symposium on Human Identification in Las Vegas, NV, sponsored by the Promega Corporation (50).

## Methods

All work for this study was conducted under the Penn State University internal review board (IRB) approved project number, 32047. A total of 33 samples were taken from related and unrelated individuals in the form of buccal cells and whole blood. Each sample was labeled according to whether it was from a man or woman, and the order in which it was collected. A total of 17 men and 16 women were collected, representing 27 different maternal lineages. Male (M) samples were given the following tracking numbers: M1-5, M7-15, M17-19 (M1 and M2 were not tested for this study). Female (F) samples were given the following tracking numbers; F1-5, F7-10, F12-13, F16, F22, F25-27 (F1 was not tested for this study). Genomic DNA was extracted from all samples using an organic-based procedure, followed by quantification of human nuclear DNA using the Applied Biosystems Quantifiler® assay (Foster City, CA, USA). Routine methods of PCR amplification and Sanger DNA sequencing were performed to determine the mtDNA sequence profile for each sample (51).

**Figure 1:**
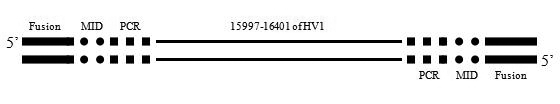
First round polymerase chain reaction (frPCR) primer design. The squares represent the primer binding sequence, the circles represent the multiplex identifier (MID) sequence, and the thick line represents the fusion primer sequence for 454 applications. The length of each primer is 49 nucleotides. The total amplicon length is 503 bps; 49 nucleotides of forward and reverse primers +405 bps of hypervariable segment 1 (HV1) representing target sequence between nucleotide positions 15 997 and 16 401.

This study focused exclusively on the HV1 of the control region. The primers used for first round PCR (frPCR) amplification flanked the entire HV1 region (nucleotide positions 16 024-16,365) and were designed to include the primer-binding sequence, a multiplex identifier (MID) sequence used to identify different samples being run together on the same plate, and a Fusion Primer sequence for binding of amplicon molecules to DNA capture beads, emulsion PCR (emPCR), and the pyrosequencing reaction (Figure 1). Five different primer sets with 5 different MIDs were designed and used for all frPCR amplifications (Table 1).

**Table 1:** First round polymerase chain reaction (frPCR) primer sequences for hypervariable segment 1. Starting at the 5′-end of each primer, fusion primer sequence for forward and reverse emulsion PCR and pyrosequencing is in standard font, followed by multiplex identifier sequence in italics and primer binding sequence in bold

Primer	Sequence
MID1 forward	5′ – GCCTCCCTCGCGCCATCAG *ACGAGTGCGT***CACCATTAG CACCCAAAGCT** – 3′
MID1 reverse	3′ – **GTGGTAGGAGGCACTTTAGT***TGCGTGAGCA* GACTCG CCCGACCGTTCCG – 5′
MID9 forward	5′ - GCCTCCCTCGCGCCATCAG *TAGTATCAGC***CACCATTAG CACCCAAAGCT** – 3′
MID9 reverse	3′ – **GTGGTAGGAGGCACTTTAGT***CGACTATGAT* GACTCG CCCGACCGTTCCG – 5′
MID10 forward	5′ - GCCTCCCTCGCGCCATCAG *TCTCTATGCG***CACCATTAG CACCCAAAGCT** – 3′
MID10 reverse	3′ – **GTGGTAGGAGGCACTTTAGT***GCGTATCTCT* GACTCG CCCGACCGTTCCG – 5′
MID11 forward	5′ – GCCTCCCTCGCGCCATCAG *TGATACGTCT***CACCATTAG CACCCAAAGCT** – 3′
MID11 reverse	3′ – **GTGGTAGGAGGCACTTTAGT***TCTGCATAGT* GACTCG CCCGACCGTTCCG – 5′
MID12 forward	5′ – GCCTCCCTCGCGCCATCAG *TACTGAGCTA***CACCATTAG CACCCAAAGCT** – 3′
MID12 reverse	3′ – **GTGGTAGGAGGCACTTTAGT***ATCGAGTCAT* GACTCG CCCGACCGTTCCG – 5′

The quantity of input DNA from each sample for frPCR was 20 ng, based on quantification of nuclear DNA. Therefore, total mtDNA quantities were unknown, and actual mixture ratios were determined empirically. Each amplification included 1 × PCR buffer, 10 mM dNTPs, 2.5 units of AmpliTaq Gold (all from Applied Biosystems), and 10 pmols of each primer (Integrated DNA Technologies, Coralville, Ia, USA). Cycling conditions for all amplifications were a 95°C soak for 10 minutes followed by 28 cycles of 94°C for 1 minute, 60°C for 1 minute, and 72°C for 1 minute. Following frPCR, the concentration of mtDNA amplicon was determined for each sample by running 20% of the product on a 2% agarose gel and comparing the band intensities to known standards. Each sample was then diluted to an approximate concentration of 1 × 10^9^ molecules/μL using a formula provided in the 454 Life Sciences protocol (49). The typical dilution factor was 10-100, or 1-2 orders of magnitude. Therefore, it is possible that 0.2-2 ng of input nuclear DNA would suffice for analysis. Experiments to test this supposition are being conducted, as ultimate levels of sensitivity will be an important factor for making the method operational. Once each sample was diluted to 1 × 10^9^ molecules/μL, 10 μL or 10 × 10^9^ molecules were pooled for each sample containing a different MID. For the 5 samples run in each experiment, this resulted in a total volume of 50 μL. A total of 2 μL of this pooled set of amplicons was diluted further by combining the 2 μL with 198 μL of dionized water, or diluted by an additional factor of 100. Again, this additional dilution step could be eliminated to further reduce the amount of input DNA used for the frPCR. The pooled and diluted amplicons were then run together through the emPCR and pyrosequencing steps of the process.

All samples were processed using the 454 GS Junior Titanium Series Lib-A emPCR Kit. This kit allows for the analysis of both strands of the amplicon, enabling the sequence data to be confirmed in both directions. Following emPCR, the capture beads with bound DNA were enriched. The enrichment process (performed according to the 454 GS Junior manual) (49) uses a second DNA capture mechanism to separate out beads with and without bound emPCR products. This process maximizes the capacity of useful beads used in the pyrosequencing step. The quantity of enriched beads was estimated, and if the output was between 500 000 and 2 million beads, the experiment was deemed a success. The enriched pool of beads was used for pyrosequencing on the GS Junior instrument, performed according to the 454 GS Junior manual (49). Analysis of the resulting mtDNA sequence data was performed using the NextGENe^®^ software package from SoftGenetics, Inc (State College, PA, USA). Specific modifications were made to the software to address the analysis of forensic mtDNA polymorphisms; eg, comparison to the Cambridge Reference Sequence (CRS, 25).

By using the MID system to differentiate samples being run together on the same pyrosequencing plate, a total of 44 different experiments were performed for this study over 9 instrument runs. The first 15 experiments (3 instrument runs) were used to determine the threshold for detecting mixture components. Two individuals were selected for each run who did not share mtDNA sequence polymorphisms in HV1; the 3 pairs of samples were F12/M15, M9/M11, and M15/F22. Mixtures of the 2 individuals were made in ratios of 1:5, 1:100, 1:250, 1:500, and 1:1000; based on nuclear DNA quantification values. The samples for each of the 5 ratios were mixed together pre-frPCR, and primers with different MIDs were used so that the 5 mixtures could be run together on the GS Junior. Each set of resulting data was partitioned with the NextGENe^®^ software by using the coded MID sequences, and aligned with the CRS. Forensic polymorphisms were identified on the actual sequence as shaded nucleotides, and listed in a table format with other data, such as the percentage of each polymorphism and the number of sequencing reads in the forward and reverse directions. Analysis was performed on the mixture samples with filters set extremely low to allow for all variants to be evaluated. However, we determined that single nucleotide differences had to be observed at least 20 times to be considered reliable during the analysis phase.

A total of 20 experiments (4 instrument runs) were performed on 20 unrelated individuals with 19 different HV1 haplotypes. One of the 20 samples failed to give acceptable levels of frPCR product. Therefore, only 19 of these samples produced reportable data. These 19 samples were run to assess the 454 system’s ability to detect previously unseen low-level heteroplasmic variants when compared to Sanger sequencing results. To test reproducibility, 4 experiments (a single instrument run) were performed. Two samples that had been previously analyzed were run in duplicate (F5 and M10), and differentiated with the use of 4 MIDs; M15 was also run in duplicate in the mixture studies. These experiments allowed for the assessment of the system’s reproducibility when detecting low-level heteroplasmic variants, and to determine whether the variants were, in fact, truly heteroplasmy as opposed to PCR and/or sequencing artifacts. The last 5 experiments (a single instrument run) involved a comparison of mtDNA sequence data from 5 maternal relatives. The 5 relatives were a grandmother, a daughter and two sons of the grandmother, and a granddaughter of the grandmother. The mtDNA sequence data for these 5 individuals was compared to assess potential differences in minor variants when the major sequence is identical.

A single instrument run was conducted by 454 Life Sciences in Branford, CT, USA, using primers designed for forensically relevant STR (D18S51) and Y-STR loci (DYS389 I/II), along with primers for the latter half of HV1, or what has traditionally been referred to as primer set 2 (PS2) (Table 2). Sequence data was generated on the 454 GS FLX Titanium instrument. For this experiment, the plate was divided into 8 different channels for 8 individual experiments. The first 3 experiments involved 3 different individuals; one female, F1, and two male, M1-2. Experiments 4 and 5 were post-PCR mixtures of a 1:1 ratio of two different samples amplified with different MIDs. These experiments served as a test for the ability of the instrument and the software to partition the data. The final 3 experiments involved pre-PCR mixtures of two individuals (M1 and M2), in ratios of 1:1, 1:5, and 1:20, to illustrate that conventional forensic mixtures could be identified and resolved for STR, Y-STR, and the PS2 amplicons.

**Table 2:** First round polymerase chain reaction (frPCR) primer sequences for primer set two (PS2) of hypervariable segment 1, the D18S51 short tandem repeat locus, and the DYS389 I/II loci. Starting at the 5′-end of each primer, fusion primer sequence for emulsion PCR and pyrosequencing is in standard font, followed by multiplex identifier sequences in italics and primer binding sequence in bold

Primer	Sequence
PS2 MID1 forward	5′ – GCCTCCCTCGCGCCATCAG *ACGAGTGCGT***ATACTTGA CCACCTGTAGTAC** – 3′
PS2 MID1 reverse	3′ – **GTGGTAGGAGGCACTTTAGT***TGCGTGAGCA* GACTCG CCCGACCGTTCCG – 5′
PS2 MID11 forward	5′ – GCCTCCCTCGCGCCATCAG *TGATACGTCT***ATACTTGA CCACCTGTAGTAC** – 3′
PS2 MID11 reverse	3′ – **GTGGTAGGAGGCACTTTAGT***TCTGCATAGT* GACTCG CCCGACCGTTCCG – 5′
D18S51 MID1 forward	5′ – GCCTCCCTCGCGCCATCAG *ACGAGTGCGT***TGACAAA TTGAGACCTGTCTC** – 3′
D18S51 MID1 reverse	3′ – **GAGGTGTGTGGTCTCTTCAA***TGCGTGAGCA* GACTCG CCCGACCGTTCCG – 5′
D18S51 MID11 forward	5′ – GCCTCCCTCGCGCCATCAG *TGATACGTCT***TGACAAA TTGAGACCTGTCTC**– 3′
D18S51 MID11 reverse	3′ – **GAGGTGTGTGGTCTCTTCAA***TCTGCATAGT* GACTCG CCCGACCGTTCCG – 5′
DYS389 I/II MID1 forward	5′ – GCCTCCCTCGCGCCATCAG *ACGAGTGCGT***AACTCTCA TCTGTATTATCTATG** – 3′
DYS389 I/II MID1 reverse	3′ – **CCCTATTAACACAAGGAGTTC***TGCGTGAGCA* GACTCG CCCGACCGTTCCG – 5′
DYS389 I/II MID11 forward	5′ – GCCTCCCTCGCGCCATCAG *TGATACGTCT***AACTCTCA TCTGTATTATCTATG**– 3′
DYS389 I/II MID11 reverse	3′ – **CCCTATTAACACAAGGAGTTC***TCTGCATAGT* GACTCG CCCGACCGTTCCG – 5′

## Results

The HV1 mtDNA sequence profile for 25 of the 27 lineages analyzed in the current study was generated using both the traditional Sanger method and the 454 GS Junior SGS approach (Table 3); the PS2 sequence of samples F1, M1, and M2 was previously evaluated in a pilot study (data not shown), although a maternal relative of F1 was run with the current study (F9). A total of 4% of the lineages (1 out of 25) showed reportable heteroplasmy with the Sanger method compared to 44% (11 out of 25) when using the SGS approach. A combined total of 15 different nucleotide positions showed minor component heteroplasmy: 16093 (T^A^), 16111 (C^A^), 16126 (T), 16128 (T), 16129 (G), 16192 (C^A^), 16209 (C), 16222 (T), 16223 (C^A^), 16261 (C^A^), 16278 (C^A^), 16293 (G), 16298 (T^A^), 16304 (T^A^), and 16311 (C and T^A^), with the **A** in X**^A^** annotating that the minor variant is the same as the CRS. Many of the sequence positions are consistent with mutational hot spots or positions where forensic polymorphisms and heteroplasmy have been observed in the past (10,16). A total of 19 observations of heteroplasmy were made at these 15 nucleotide positions for the 25 lineages; duplicates were seen at 16093 (T), 16192 (C), 16304 (T), and 16311 (C and T).

**Table 3:** The hypervariable segment 1 mitochondrial DNA (mtDNA) profiles for 25 of the 27 lineages; polymorphic nucleotide sites are in relation to the Cambridge Reference Sequence. The percentage of minor component heteroplasmy at specific sites is provided for each sample, when detected

Sample	Sanger mtDNA profile	Percent of minor heteroplasmy and site	454 GS Junior mtDNA profile	Percent of minor heteroplasmy and site
F2	16069T, 16093C, 16126C, 16261T, 16274A, 16355T	16311 – 18.4% C	16069T, 16093C, 16126C, 16261T, 16274A, 16355T	16093 – 3.71% T 16261 – 1.29% C 16311 – 20.14% C
F3	16069T, 16126C, 16145A, 16172C, 16261T	Not detected	16069T, 16126C, 16145A, 16172C, 16261T	Not detected
F4	No polymorphisms	Not detected	No polymorphisms	Not detected
F5	16129A, 16172C, 16223T, 16311C	Not detected	16129A, 16172C, 16223T, 16311C	16129 – 0.51% G 16311 – 0.33% T
F7, F12-13, M13-14	16192T, 16256T, 16270T	Not detected	16192T, 16256T, 16270T	16192 – 2.64%-4.50% C
F8	16223T, 16362C	Not detected	16223T, 16362C	16223 – 1.86% C
F9	16356C	Not detected	16356C	Not detected
F10	16298C	Not detected	16298C	16298 – 0.45% T
F16	16126C, 16239T, 16294T, 16296T, 16304C	Not detected	16126C, 16239T, 16294T, 16296T, 16304C	Not detected
F25	16343G	Not detected	16343G	Not detected
F26	16093C	Not detected	16093C	Not detected
F27	16172C, 16278T	Not detected	16172C, 16278T	Not detected
M3	16355T	Not detected	16355T	Not detected
M4	16111T	Not detected	16111T	16111 – 0.52% C
M5	16114A, 16129A, 16192T, 16213A, 16223T, 16278T, 16355T, 16362C	Not detected	16114A, 16129A, 16192T, 16213A, 16223T, 16278T, 16355T, 16362C	16192 – 3.18% C
M7	16129A, 16223T, 16264T	Not detected	16129A, 16223T, 16264T	Not detected
M8	16224C, 16311C	Not detected	16224C, 16311C	Not detected
M9	16301T, 16343G, 16356C	Not detected	16301T, 16343G, 16356C	Not detected
M10	16304C	Not detected	16304C	16209 – 2.62% C 16222 – 2.30% T 16304 – 2.99% T
M11	16129A, 16223T	Not detected	16129A, 16223T	Not detected
M12	16069T, 16126C	Not detected	16069T, 16126C	16126 – 1.14% T
M15	16093C, 16224C, 16311C	Not detected	16093C, 16224C, 16311C	16093 – 3.04% T
M17	16126C, 16294T, 16296T	Not detected	16126C, 16294T, 16296T	Not detected
M18	16278T, 16304C, 16311C	Not detected	16278T, 16304C, 16311C	16128 – 0.52% T 16278 – 0.77% C 16293 – 0.77% G 16304 – 1.00% T
M19, F22	16069T, 16126C, 16222T	Not detected	16069T, 16126C, 16222T	Not detected

**Figure 2:**
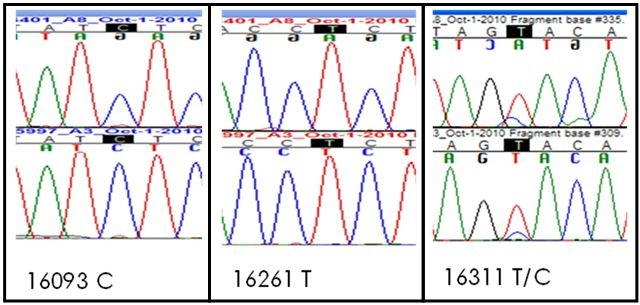
Heteroplasmy at positions 16 093, 16 261, and 16 311 of sample F2, as observed when using the Sanger method of DNA sequencing. The only position where heteroplasmy would have been called by the majority of forensic scientists is 16 311; laboratories using instruments with low levels of signal to noise may have called position 16 093. The corresponding percentages of heteroplasmy observed in the second generation sequencing data were, 3.71%, 1.29%, and 20.14%, respectively.

Only one of the 25 lineages (F2) exhibited reportable heteroplasmy for both of the sequencing methods. This sample had approximately 20% T/C heteroplasmy at position 16311, which was easily identified by the Sanger method (Figure 2); the T/C designation indicates that the T is major and the C is minor. Unfortunately, the only approach for providing meaningful estimates of the percentage of heteroplasmy in Sanger data are a physical measurement of peak heights. For sample F2, the height of the T peak at 16311 was 10 mm on average (using forward and reserve data), whereas the average height of the underlying C peak was 2.25 mm. Therefore, the amount of minor component C in the T/C heteroplasmy could be *estimated* at 18.4%. Conversely, the SGS data *measured* the amount of the minor component C at 20.14% by dividing the number of C-reads by the total reads. For all other observations of heteroplasmy in the remaining 24 lineages, the minor component did not reach a level that was, or typically would be, observable with the Sanger method (Figure 2); all minor component values were within a measured range of 0.33% to 4.50%, or in the range of mixture ratios of 1:20 to 1:300. In addition, all of the low-level heteroplasmic positions had coverage rates of at least 40 reads, with most having well over 100 reads. Therefore, these results can be considered highly reliable.

The reproducibility of detecting minor component heteroplasmy was evaluated by looking at two samples in triplicate (F5 and M10), and one sample in duplicate (M15). The first sample (F5) had low-level heteroplasmy at positions 16129 A/G and 16311 C/T. The percentages of heteroplasmy for each position were relatively consistent between and within experiments; 0.51%, 1.06%, and 0.36% for 16129 (an average ratio of 1:155), and 0.33%, 1.09%, and 1.82% for 16311 (an average ratio of 1:93). The second sample (M10) had low-level heteroplasmy at 16209 T/C, 16222 C/T, and 16304 C/T. The percentage of heteroplasmy for each position was, again, relatively consistent between and within experiments; 2.62%, 2.58%, and 2.32% for 16209 (an average ratio of 1:40), 2.30%, 2.03%, and 2.57% for 16222 (an average ratio of 1:44), and 2.99%, 1.87%, and 0.56% for 16304 (an average ratio of 1:55). The last sample (M15) had low-level heteroplasmy at 16093 C/T. The percentage of heteroplasmy for this position was, yet again, consistent between and within experiments; 3.04% and 3.40% for an average ratio of 1:30. Therefore, based on these data, the SGS method as it was employed in this study is reproducible when detecting low-level heteroplasmy variants in an approximate range of 0.33%-2.99%. Given the robustness of the data, and the relatively low percentage of heteroplasmy being detected, we can assume that the method will remain reproducible at higher percentages of heteroplasmy.

The total number of sequence reads (ie, the coverage rate), along with the percentage of minor component sequence or the number of minor component sequence reads, and the distribution of minor component reads in both the forward and reverse direction, allow for a general assessment of the actual mixture ratio in comparison to the estimated ratio that was based on nuclear DNA quantification data (Table 4). As expected, the actual ratios were, in some cases, quite different from the estimated values. In some cases, these values were quite lower than expected, which further enhances the value of the data. These data can also be used to assess the level of reliability for identifying minor components at the different ratio levels based on coverage rates and the confirmation of sequence data on both strands of DNA. For example, the minor component was easily distinguishable in all 6 mixtures with a ratio of 1:5 (20%) and 1:100 (1%), as the total number of minor reads ranged from 21 to 1906, and the ratio of forward to reverse reads was comparable to the ratio of total reads. The actual values for these two target ratios ranged from 1:6 to 1:8 for the 1:5 mixture, and 1:116 to 1:178 for the 1:100 mixture; the second mixture experiment yielded considerably lower read numbers, so these data were interpreted with caution.

**Table 4:** A summary of the data for the 3 sets of 5 mixture experiments at the estimated ratios of 1:5, 1:100, 1:250, 1:500, and 1:1000. Total coverage is the total number of sequencing reads generated by the instrument; minor component percentage is the percentage of the total reads that correspond to the minor nucleotide; minor component coverage is the total number of sequencing reads generated by the instrument for the minor component; forward:reverse sequence reads is the total number of sequencing reads generated by the instrument in the forward and reverse directions; and minor vs total is a reflection that the number on the top ratios are minor (eg, 662:363) and the bottom ratios are total (eg, 5425:2946)

	Experiment 1 (F12:M15)		Experiment 2 (M9:M11)		Experiment 3 (M15:F22)	
**Mixture**	**total coverage**	**minor component percentage**	**total coverage**	**minor component percentage**	**total coverage**	**minor component percentage**
1:5	8371	12.24	1950	7.18	11310	16.85
1:100	11126	0.56	2751	0.11	2436	0.86
1:250	7241	0.36	2830	0.11	3959	0.38
1:500	9941	0.24	7357	0.07	25705	0.051
1:1000	5848	0.07	1234	0.16	2991	0.100
	minor component coverage	forward: reverse sequence reads minor vs total	minor component coverage	forward: reverse sequence reads minor vs total	minor component coverage	forward: reverse sequence reads minor vs total
1:5	1025	662:363 5425:2946	140	122:18 1651:299	1906	1181:725 7492:3818
1:100	62	46:16 7910:3216	3	3:0 2410:341	21	12:9 1783:653
1:250	26	19:7 4789:2452	3	2:1 2329:501	15	9:6 2490:1469
1:500	24	14:10 6945:2996	5	5:0 7012:345	13	8:5 18411:7294
1:1000	4	3:1 3954:1894	2	1:1 972:262	3	2:1 1971:1020
	actual mixture ratio		actual mixture ratio		actual mixture ratio	
1:5	1:8		1:14		1:6	
1:100	1:178		1:917		1:116	
1:250	1:278		1:917		1:264	
1:500	1:417		1:1428		1:1977	
1:1000	1:1428		1:625		1:997	

In general, the ratio of forward to reverse reads for minor component sites when compared to the total was quite consistent. For example, the average ratio of forward to reverse reads for the two minor component sites observed in sample F5 was 2.66, compared to 2.75 for the total ratio. The difference between these two values is 3.3%. A similar result was seen for the average ratio of forward to reverse reads for the 3 minor component sites observed in sample M10; 1.76 vs 1.84, 4.4%. However, there was variability observed in the ratio of values for other samples, occurring most often for samples containing a single minor component site. For example, the ratios for sample M12 gave the highest differences, 6.86 (minor) vs 2.20 (total), for a difference of 212%. While this is clearly a significant difference in a mathematical sense, the number of total reads for the minor component site in this sample was 165, the percentage of the minor variant was 1.14%, and this is a known site where sequence difference occur between individuals, so it is unlikely that this observation was due to a PCR or sequencing error.

While the reproducibility experiments were useful in addressing the reliability of reported data, we did an assessment of potential PCR and sequence errors in an attempt to establish thresholds and/or filters. For the current study, insertions and deletions were not considered. Sequence changes (forensic polymorphisms), in relation to the CRS, were captured and assessed individually. The following considerations were made: 1) the change was not seen in other non-related data conducted during the same run, 2) the change was plausible based on the actual sequence data, looking for things like miss alignment, 3) the change was seen more than 40 times, 4) the ratio of forward to reverse reads was consistent when comparing the minor site ratio to the total value, and 5) the change was a transition vs a transversion. If two or more of these criteria were violated, the change was not reported. Interestingly, in one experiment (samples from 5 different individuals), we observed sequence changes at the following 7 positions in all 5 samples: 16042 T, 16095 A, 16145 T, 16148 A, 16174 A, 16250 A, and 16328 A. Each of these changes is also a transversion from the CRS, and in some instances violated at least one other criterion as stated above. Therefore, these changes were not reported. It is unclear whether these were PCR or sequencing errors, although since none of these 7 changes was seen in other samples in other runs (including for runs conducted with the same reagent lot number), it seems likely that these were PCR-based errors for this one set of data.

For the 1:250 mixtures, the minor component was easily detected when the coverage rate was at least 5000, but was still readily observed even when coverage dropped to 2830 reads. The actual ratios for experiments 1 and 3 were 1:278 and 1:264, respectively. These data were supported by the number of minor reads, and the ratio of forward to reverse reads when compared to the ratio of total reads. The ability to detect minor component sequences in 1:500 and 1:1000 mixtures was dependent on coverage rate (ideally at least 10 000 and 20 000, respectively), which was not met in most instances during this study. Only twice in the 6 experiments did the coverage reach or approach 10 000; 9941 in experiment 1 and 25 705 in experiment 3, both for the 1:500 mixture ratio. In experiment 1, the number of minor reads reached 24, and resulted in a measured mixture ratio of 1:417. In addition, the ratio of forward to reverse reads was consistent with the total read ratio. However, in experiment 3, the number of minor reads only reached 13, even with more than 25 000 total reads, for a measured ratio of 1:1977. Therefore, without greater coverage, it is difficult to reliably report minor components in mixture ratios of 1:500 to 1:1000.

The family study yielded results with limited value. All 5 members had the 16192 T, 16256 T, and 16270 T forensic polymorphisms. In addition, they each had SGS-detectable levels of heteroplasmy at position 16192 (C). The range of heteroplasmy was 2.64%-4.50%, so this position was not able to differentiate individuals within the same lineage. In addition, very low-level heteroplasmy was detected at position 16129 (A/G), but too low to be considered reliable enough to report; the grandmother and mother had 0.14%-0.48% heteroplasmy, but only 3-7 reads (3/0 to 4/3, forward/reverse), and the grandchildren had 0.08%-0.31% heteroplasmy, with 4-7 reads (3/1, 4/0, and 5/2). If this heteroplasmy was, in fact, shared by all family members, it too would not be a useful position for discriminating between members.

**Figure 3:**
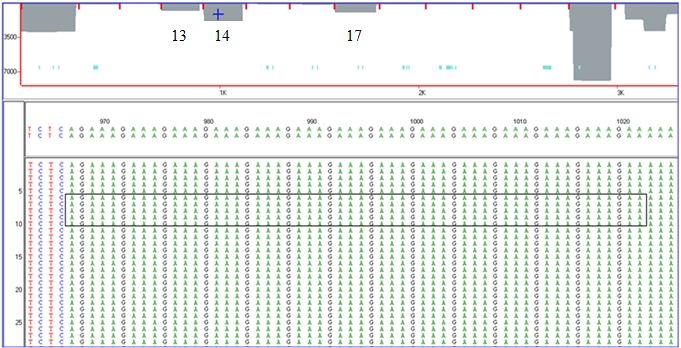
Short tandem repeat results for the D18S51 locus of two samples mixed in a pre-polymerase chain reaction ratio of 1:1. The D18 profile for individual one was 13,14 and 14,17 for individual two. The 3 gray-shaded areas labeled in the figure are representative of the sequence density for alleles 13, 14, and 17; ~ 1000-2500 sequences for each allele. The cross above the 14 allele indicates which sequence is being displayed in the window below; sequences 1-28 for allele 14. The sequence density for allele 14 is illustrative of 2 doses of that allele. The area enclosed by the box in the window below is the 14 copies of the STR repeat, AGAA, for the 14 allele; for sequences 6-10. The numbered sequence above the window is a reference used to partition the sequences associated with the 14 allele away from other alleles.

The amplicons for STR, Y-STR, and mtDNA loci could be pooled together using the MID system, their sequences generated in a single instrument run, and the resulting data partitioned into different samples, different loci and different allelic “bins.” The PCR primer binding sequence functions as the locus identifier, the MID serves as the sample identifier, and the fusion sequence is used for the pyrosequencing process. Figure 3 is an illustration of the ability of the NextGENe software to partition the allelic data for D18S51 through comparison and alignment to reference alleles. The data in Figure 3 also illustrate that conventional mixtures can be interpreted by using read numbers. This is analogous to using relative fluorescence units for conventional STR analysis. The “profile” in the figure is from a mixture of 2 individuals in an estimated ratio of 1:1. The measured ratio was 1:0.92. The data for the 1:5 and 1:20 mixtures were consistent with these findings (data not shown). The advantage of using SGS to generate STR results is that both length-based and sequence-based information can be captured at the same time. In addition, since PCR fragment size is eliminated as a factor in the analysis of the amplicon, the number of target loci can be greatly increased when compared to conventional capillary electrophoresis methods of STR analysis.

**Figure 4:**
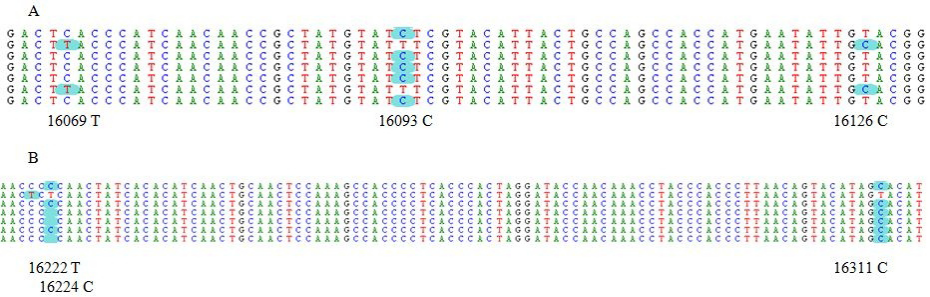
A chimeric mitochondrial DNA sequence for a sample from a 1:100 mixture of M15 and F22. The second sequence from the top has the 16069 T, 16126 C, and 16222 T polymorphisms of the minor component; highlighted by the NextGENe software. The second sequence from the bottom is a chimeric product that contains the 16 069 T and 16 126 C from the minor component, and the 16 224 C and 16 311 C polymorphisms from the major component. This is referred to as a Minor Front, Major Back chimeric sequence. The missing C’s at position 16 224 for the 4th, 5th, and 7th sequences listed below are indicative of the difficulties of the 454 approach to resolve homopolymeric stretches of greater than 4 C’s in a row.

**Figure 5:**
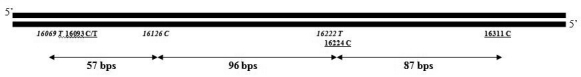
The distance between mitochondrial DNA polymorphisms in the major and minor components of a 1:5 or 1:100 mixture of M15 and F22. The major component profile from M15 has 16093 C/T heteroplasmy, 16224 C, and 16311 C, and the *Minor* component profile from F22 has *16069 T, 16126 C, and 16222 T*.

Finally, during the course of completing the recent work on this project, it was determined that chimeric mtDNA sequences were being produced through jumping PCR. This was presumably happening during the frPCR process, however, we have not ruled out the emPCR step. Data for the M15/F22 mixtures, in ratios of 1:5 and 1:100, serve as an illustration of this phenomenon (Table 5). An example of the chimeric data shows two interesting trends (Figure 4). First, when the minor component is in higher percentages (1:5), there is less jumping PCR observed. Second, if the data are “pooled,” as happens during Sanger sequencing, the percentage of each component is consistent with expected values. The distance between the polymorphisms for both M15 and F22 helps to explain the data (Figure 5). It is more likely for “recombination” like events during jumping PCR to occur between 16126 C and 16222 T, and between 16224 C and 16311 C, than between 16069 T and 16126 C. The data supports this prediction (Table 5).

**Table 5:** A summary of the complete and chimeric sequences observed in the 1:5 and 1:100 mixtures for samples M15 (major) and F22 (minor). The front and back designations reflect the first and second halves of hypervariable segment 1, respectively. The mtDNA sequence for the major and minor components can be found in Table 3 and Figure 4

Samples M15 and F22	1:5 ratio	1:100 ratio
Total sequences analyzed	1028 (of 11 310)	2436
Complete minor sequences	130	6
Major front, minor back sequences	21	5
Minor front, major back sequences	22	9
069T + major sequences	6	2
069C+ minor sequences	23	9
Major +311T sequences	16	2
Minor +311C sequences	4	2

## Discussion

The SGS platform of 454 Life Sciences, including the GS Junior instrument, produces reliable and reportable results for the analysis of mtDNA sequence data. As we reported here, the forensic mtDNA sequence profile of HV1 was correctly analyzed for 25 different maternal lineages, across 30 different individuals. In addition, the results clearly illustrate the added levels of observed heteroplasmy that can be achieved when using the SGS approach. When using the conventional Sanger dideoxy-terminator method, only one of these 25 lineages exhibited reportable heteroplasmy; a 4% rate. However, the SGS approach identified low-level heteroplasmy for 11 of the 25 lineages; a 44% rate of identifiable and reportable heteroplasmy. The nucleotide positions where heteroplasmy was observed are consistent with mutational hot spots or sites where forensic polymorphisms (in comparison to the CSR) and heteroplasmy have been observed in past studies (10,16). In particular, when the sensitivity level of heteroplasmy detection is elevated (eg, when using a denaturing gradient gel electrophoresis approach), many of the same sites of heteroplasmy identified in the current study have been previously reported (16).

The distribution of heteroplasmic sites spanned the entire range of HV1. In addition, there was a relatively even distribution of minor component sites that corresponded to the CRS, as opposed to those that differed from the CRS; 9 observations vs 7, respectively. This is an important observation when attempting to differentiate individuals with the same primary haplotype, as it may be expected that low-level heteroplasmy would exist at polymorphic positions. However, minor sequences with non-CRS nucleotides could be the result of widespread lineage-based heteroplasmy, or could be de novo changes in the sequence that occurred during early fetal development. For relatives within the same lineage, one might expect that the chances are more likely that non-CRS variants would be shared by other family members. While the family data generated for our study were not illustrative of the notion, the overall rate of low-level heteroplasmy detection reported in Table 3 suggests that it would be possible to differentiate between family members. Further studies are needed to explore a greater number of lineages, and sequence additional family members within the same lineage. Nonetheless, when faced with two reference sources in a forensic case, each of which has the same control region sequence, the prospects for tapping into low-level heteroplasmy to differentiate the two are high.

Any concerns regarding the reliability of the sequence data generated with the 454 GS Junior SGS approach were at least partially addressed through the reproducibility studies reported here. Multiple samples were run in either duplicate or triplicate, with results that confirmed the positions of heteroplasmy, and at very similar component percentages. Therefore, PCR and/or sequencing artifacts were ruled out as the source of the relatively high rate of heteroplasmic activity. In addition, the coverage rates and total number of reads for all heteroplasmic variants reported in Table 3 were high. In particular, all reported instances of low-level heteroplasmy resulted from at least 40 reads of sequence (most with more than 100 reads), and with a balanced ratio of forward and reverse reads when compared to the total ratio of forward and reverse reads. These data and observations allow for the development of initial standards for reporting low-level heteroplasmic variants in a forensic setting.

Based on the results from this study, we would recommend reporting low-level variants when at least 40 reads have been generated, and when the ratio of forward to reverse reads is consistent with the total ratio. Therefore, for mixtures of 1:100, 1:250, 1:500, and 1:1000, the coverage would need to be at least 4000, 10 000, 20 000, and 40 000, respectively. This level of coverage is well within the capability of the GS Junior instrument. However, a desire to increase coverage rates would drive down throughput and drive up costs. The reagent cost per run is currently less than US $1000, and the instrument capacity ranges from 50 000-100 000 reads per run. Therefore, if detection of low-level variants from ratios of 1:1000 were desired, the capacity per run would be 1-2 samples, with a reagent cost of less than US $500-1000 per sample. If a ratio of 1:250 were suitable, the number of samples per run would go up to 5-10, and the cost per sample would drop to less than US $100-200. Given that multiple amplifications would be needed to cover the entire control region, and including the potential for replicate testing, the total cost per sample could run as high as US $800-1600. In addition, the overall throughput of the system is lower than conventional methods currently in place. Nonetheless, the significantly increased level of heteroplasmy detection is enough of a value-added feature to make this an attractive approach when attempting to increase discrimination potential or differentiate between individuals with the same primary haplotype.

The results of the mixture study further validate that the 454 GS Junior can reliably detect minor component variants. Mixtures, including heteroplasmic variants, were detected routinely down to a component ratio of 1:250. In doing so, at least 40 minor variant copies were observed with a coverage rate of at least 5000 sequences. However, the concept of resolving mixtures is quite different than identifying low-level variants. The goal for the latter is to identify low-level single nucleotide variants below the surface of a primary sequence profile. For a mixture, the goal is to resolve individual mtDNA haplotypes from one another despite the ratio of the 2 (or more) components found in the mixture. Following this logic, while the read number, the ratio of forward to reverse reads, and the overall coverage are all directly proportional to the success of the typing approach, read number in relation to coverage rates for mixtures could be more important. Of course, mixture analysis is also affected by the introduction of foreign sources of DNA during the analysis process, which is especially relevant when considering SGS data. Historically, forensic mtDNA laboratories using the Sanger DNA sequencing method have been able to measure the rate of contamination in negative controls; as high as 5%. With SGS, it is anticipated that this rate will increase; approximately 10% in our hands. However, the effects of this concern will be dependent on where the thresholds are set for acceptance of minor component sequence as reportable data. Therefore, given the capacity and throughput of the GS Junior, and our current understanding of the results generated from this instrument, minor ratios above 1:100 would be desirable for confidently resolving mixture components.

To date, only one operational system has emerged that can resolve mtDNA mixtures (39,41). The electrospray ionization mass spectrometry (ESI-MS) approach from IBIS Biosciences is capable of resolving mtDNA mixtures with component ratios as low as 1:20 (41), and has been employed in a number of forensic laboratories performing mtDNA analysis across the United States. The principle differences between the ESI-MS and SGS systems are an inability of the ESI-MS to identify the exact location where a sequence difference exists, and the inability of the SGS system to fully resolve homopolymeric stretches and length heteroplasmy. The ESI-MS system generates the precise molecular mass of each pool of amplicons to identify species with different sequence content. However, the location of sequence differences can only be implied. In contrast, the pyrosequencing approach of the 454 SGS system results in primary sequence information, but provides poor resolution of homopolymeric sequences, and reveals PCR and sequencing artifacts that will require a filtering mechanism to eliminate their consideration during analysis; similar to filters needed for STR stutter and spectral bleed. Therefore, at this point in time, neither system can provide the complete information necessary to make precise interpretations across the entire control region. However, given the complexities of mtDNA mixture deconvolution, the SGS approach may be more desirable, since it provides primary sequence. In addition, the ESI-MS approach from IBIS Biosciences, while commercially available, would be cost-prohibitive for most forensic laboratories, as both the instrument and reagent costs are high, and the throughput is approximately the same as the GS Junior. Nonetheless, either approach allows for the deconvolution of mtDNA mixtures, which has not been available in the past.

The strengths and limitations of the discrimination potential of conventional mtDNA testing have been well documented (2). Increasing the discrimination level has been attempted by looking outside of the HV1 and HV2 regions, and expanding the scope of analysis to the coding region (for example, 26,29). However, it has also been well documented that tapping into the underlying levels of heteroplasmy in the control region is an excellent method for enhancing the discrimination of the typing system (13). In the identification case of Nicholas Romanov, the presence of heteroplasmy in the Romanov family allowed for the calculation of a likelihood ratio (LR) that took both the haplotype frequency **and** the probability of observing heteroplasmy at any one position along the mtDNA control region into account. The LR for identity of the Tsar when based strictly on the haplotype of the skeletal remains was calculated as 150, and the LR for the presence of a heteroplasmic sequence shared by two brothers was calculated as 2500 (Nicholas and Georgij Romanov). Therefore, since these two events were considered to be independent of one another, the LR for the mtDNA evidence was 375 000 more likely if the remains were, in fact, those of Nicholas Romanov. Similar calculations could be generated in forensic cases to assess the weight of observing low-level heteroplasmic variants that are shared by the evidence and a reference profile.

The presence of chimeric mtDNA sequences in our data, presumably produced through jumping PCR, was not an unexpected occurrence. This has been observed in a number of instances in other laboratories (52-54, and personal communication from Walther Parson, Innsbruck, Austria), mainly when dealing with ancient DNA. Methods such as single-molecule PCR (smPCR) have distinct advantages over conventional vector-based cloning techniques or SGS, as they avoid PCR-related artifacts, preferential allelic amplifications, and jumping PCR. However, in forensic investigations, this is somewhat impractical. We are in the process of investigating why the jumping PCR is occurring in our system, and more importantly, how it can be mitigated. Given that we used considerably more input DNA than necessary for the frPCR process, and that there is less jumping PCR observed when the minor component is in higher percentages (eg, 1:5), it is quite possible that by simply reducing template DNA we can eliminate jumping PCR from happening. However, if further steps are necessary, we are also evaluating how to modify the PCR conditions to generate a similar effect; for example, by increasing the time of extension or by looking at different polymerases. It will be important to address this issue if the technique is to become operational in forensic DNA laboratories. The greatest effect of the jumping PCR phenomenon will be on mixture interpretation, as this type of analysis requires the production of full length sequences from a single contributor. Nonetheless, for detection of low-level heteroplasmic variants, if the SGS data are “pooled” as happens during Sanger sequencing, the percentage of each component remains consistent with expected values.

While the future of second generation DNA sequencing in forensic cases is still under investigation, it is clear that this technology has the potential to expand the number and types of loci being analyzed together. For example, results from out pilot study clearly illustrate that sequence of amplicon targets from STR, Y-STR, and mtDNA loci can be generated at the same time. Using the expanded format of 454 Life Sciences (FLX), this could provide a cost-effective and rapid way to database offender samples for an expanded list of autosomal and lineage markers, as well as markers for human morphological features and geo-profiling; reagent costs of less than US $20 per sample for more than 50 loci of data have been made (50). Of course, the newer technologies of third generation sequencing (eg, single molecule sequencing from the Pacific Biosciences RS and the Ion Torrent PGM system) will need to be evaluated closely to determine whether they possess solutions to the limitations of the currently available systems. Hence, while it has been more than two decades since DNA testing has emerged on the scene, this is an exciting time in the continuing evolution of the forensic DNA community.
